# Molecular analysis of *Gyrovirus galga1* variants identified from the sera of dogs and cats in China

**DOI:** 10.1080/01652176.2024.2338381

**Published:** 2024-04-10

**Authors:** Shuqi Xu, Yuanzhuo Man, Zhengli Yu, Xin Xu, Jun Ji, Yunchao Kan, Yingzuo Bi, Qingmei Xie, Lunguang Yao

**Affiliations:** a Henan Provincial Engineering Laboratory of Insects Bio-reactor, Henan Provincial Engineering, and Technology Center of Health Products for Livestock and Poultry, Henan Provincial Engineering and Technology Center of Animal Disease Diagnosis and Integrated Control, Nanyang Normal University, Nanyang, PR China; b College of Animal Science, South China Agricultural University, Guangzhou, PR China

**Keywords:** G*yrovirus galga1*, dogs, cats, phylogenetic analysis, recombination analysis

## Abstract

*Gyrovirus galga1* (GyVg1), a member of the *Anelloviridae* family and *Gyrovirus* genus, has been detected in chicken and human tissue samples. In this study, the DNA of GyVg1-related gyroviruses in the sera of six dogs and three cats from Central and Eastern China was identified using PCR. Alignment analysis between the nine obtained and reference GyVg1 strains revealed that the genome identity ranged from 99.20% (DOG03 and DOG04 strains) to 96.17% (DOG01 and DOG06 strains). Six recombination events were predicted in multiple strains, including DOG01, DOG05, DOG06, CAT01, CAT02, and CAT03. The predicted major and minor parents of DOG05 came from Brazil. The DOG06 strain is potentially recombined from strains originating from humans and cats, whereas DOG01 is potentially recombined from G17 (ferret-originated) and Ave3 (chicken-originated), indicating that transmissions across species and regions may occur. Sixteen representative amino acid mutation sites were identified: nine in VP1 (12 R/H, 114S/N, 123I/M, 167 L/P, 231 P/S, 237 P/L, 243 R/W, 335 T/A, and 444S/N), four in VP2 (81 A/P, 103 R/H, 223 R/G, and 228 A/T), and three in VP3 (38 M/I, 61 A/T, and 65 V/A). These mutations were only harbored in strains identified in dogs and cats in this study. Whether this is related to host tropism needs further investigation. In this study, GyVg1 was identified in the sera of dogs and cats, and the molecular characteristics prompted the attention of public health.

## Introduction

In 2011, human gyrovirus (HGyV) was identified in the skin swabs of healthy humans, and *Gyrovirus galga1* (GyVg1, previously recognized as *Avian gyrovirus2* [AGyV2]), which shared 96% genome sequence similarity with HGyV, was first detected in diseased chickens from Brazil (Rijsewijk et al. 2011). GyVg1 strains are primarily found in chickens, which are generally considered their original hosts. However, the DNA of HGyV/GyVg1 was also detected in blood samples from organ-transplant recipients and HIV-infected individuals from Italy as well as healthy blood donors from France (Maggi et al. 2012; Biagini et al. 2013). GyVg1 DNA has been detected in humans (Sauvage et al. 2011; Ye et al. 2015), ferrets (Fehér et al. 2015), snakes (Wu et al. 2019), cats (Niu et al. 2019), dogs (Liu et al. 2022), and various zoo animals (Ji et al. 2022). The wide prevalence of GyVg1 in chickens and other animals indicates that it may threaten human health.

GyVg1 belongs to the viral genus *Gyrovirus* (family: *Anelloviridae*) and possesses a negative-sense, single-stranded, circular DNA genome with 2375/2376 nucleotides in length, comprising three overlapped open reading frames encoding VP1 (capsid protein), VP2 (nonstructural protein), and VP3 (functional proteins with apoptotic effect) (Yao et al. 2017). In chickens, GyVg1 infections can cause brain damage, mental impairment, and weight loss (dos Santos et al. 2012). As another member of the *Anelloviridae* family, *Gyrovirus chickenanemia* (GyVCA), which harbors high sequence similarity with GyVg1, causes illness in young chickens and is characterized by atrophic alterations in lymphoid organs, aplastic bone marrow, and hemorrhagic lesions in muscles. (Gimeno and Schat 2018; Fatoba and Adeleke 2019). GyVCA and GyVg1 are major sources of poultry vaccine contaminants in Brazil and China and are becoming increasingly prevalent in chickens (Varela et al. 2014; Yao et al. 2017). In China, GyVg1 was detected for the first time in a chicken flock and healthy humans in 2015 (Ye et al. 2015). Considering the widespread infection of chickens with GyVg1 and its potential transmissibility to humans, investigation of its infection status in companion animals, such as cats and dogs, would aid in understanding how GyVg1 is transmitted from chickens to humans.

GyVg1 has been detected in fecal samples of cats and serum samples of dogs (Niu et al. 2019; Liu et al. 2022). To date, GyVg1 has not been successfully isolated *in vitro*, and it remains unclear whether companion animals are infected with the virus. Identification of GyVg1 in more number of serum samples may provide more convincing evidence for its infection in these companion animals. In this study, we aimed to detect GyVg1-related gyroviruses in cats and dogs and to analyze their whole-genome sequences to reveal molecular characteristics of the evolution and mutation of GyVg1.

## Materials and methods

### Sample collection and virus screening

From 2020 to 2023, 237 serum samples (171 from dogs and 66 from cats) were kindly supplied by pet hospitals located in Hubei, Henan, Anhui, and Zhejiang provinces, China. To ensure ethical considerations, all pet owners provided informed consent, and veterinary clinicians collected all samples following the guidelines set by the South China Agricultural University Committee for Animal Experiments approved the protocols for serum sample collection (approved ID: SYXK 2019-0136, June 8, 2020).

Viral DNA was extracted from samples using the EasyPure Viral DNA/RNA Kit (TransGen Biotech, Inc., Beijing, China) following the manufacturer’s instructions. The extracted DNAs were screened for GyVg1 *via* PCR using the GyVg1-specific primer set comprising GyVg1-F (5′-CGTGTCCGCCAGCAGAAAC-3′) and GyVg1-R (5′-GGTAGAAGCCAAAGCGTCCAC-3′) modified from a previous report (Ye et al. 2015).

### Virus cultivation

Chicken embryo fibroblasts (DF-1), Leghorn male hepatoma cells (a chicken hepatoma cell line), Crandell–Rees feline kidney (CRFK) cells, and Madin–Darby canine kidney (originating from dogs) cells were incubated with GyVg1-positive sera, respectively. Cultures were microscopically screened daily for cytopathic effect (CPE) for 4 days till five blind serial passages. Culture lysates and supernatants from each passage were harvested for detection using the PCR method described above.

### Whole-genome sequencing of GyVg1

The complete genome sequences of GyVg1 were amplified from the extracted viral DNA using PCR with PrimeStar HS DNA polymerase (TaKaRa Bio Inc., Kusatsu, Japan) and three primer pairs, as described previously (Yao et al. 2016). The PCR amplicons of the three fragments were inserted into a pMD18-T easy vector (TaKaRa Bio Inc., Kusatsu, Japan) for sequencing (Sangon Biotech, Shanghai, China). All amplification and sequencing were performed at least thrice to ensure sequence accuracy.

### Sequence and phylogenetic analyses

SeqMan software (DNASTAR, Lasergene®, Madison, Wisconsin) was used for the contig-assembly of the DNA fragment. After alignment of the nine obtained sequences with the 38 reference sequences (information for the references are shown in Supplementary Table S1) available in GenBank *via* ClustalX v1.83. A phylogenetic tree of GyVg1 was constructed using Molecular Evolutionary Genetic Analysis v.11.0, with the maximum likelihood method, the Kimura 2-parameter model, and pairwise deletion, with bootstrap analysis of 1000 (Tamura et al. 2021). Referring to a previous report, the VP1 protein and the untranslated regions (UTR) contained numerous variant sites and recombination breakpoints; therefore, phylogenetic analysis for amino acid (aa) sequences of VP1 and nucleotide (nt) sequences of UTR were also conducted. The aa sequences of VP1–3 of the obtained and reference GyVg1 strains underwent multiple alignments to monitor the mutations.

### Recombination analysis

The genome sequences of all GyVg1 strains were used for recombination prediction using RDP4 4.83 software with the RDP, GENECONV, MaxChi, BootScan, and SiScan methods (Martin et al. 2015). SimPlot 3.51 software was used to evaluate and confirm all recombination occurrences (Truchado et al. 2019).

## Results

### Positive rates of GyVg1 expression in companion animals

GyVg1 was detected in 6 of 171 dogs (3.5%) and 3 of 66 cats (4.5%). Nine positive serum samples were acquired from several pet clinics in Hubei (one cat and two dogs), Henan (one cat and two dogs), Anhui (two dogs), and Zhejiang (one cat) provinces, China. Table 1 shows the clinical information about each GyVg1-positive dog and cat.

**Table 1.** Detailed information of the obtained GyVg1 stains.

Strain name	Accession no.	Year	Province	Host	Age
CAT01	OR921197	2020	Hubei	Cat	56 d
DOG01	OR921198	2021	Anhui	Dog	4 m
CAT02	OR921199	2021	Henan	Cat	18 m
DOG02	OR921200	2021	Henan	Dog	27 d
DOG03	OR921201	2022	Hubei	Dog	2 m
DOG04	OR921202	2022	Anhui	Dog	6 m
CAT03	OR921203	2022	Zhejiang	Cat	36 m
DOG05	OR921204	2023	Hubei	Dog	13 m
DOG06	OR921205	2023	Henan	Dog	7 m

### Virus cultivation

Until the fifth passage, no particular CPE was observed in the cultivated cells, and GyVg1 DNA was not detected using PCR.

### Identity analysis

**Figure 1.**
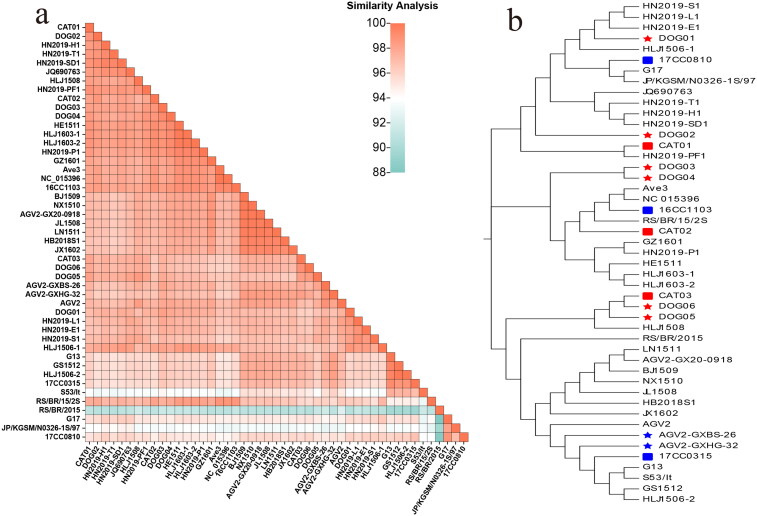
Analysis of the evolution and identity of genome nt sequences. (a) The GyVg1 strains developed in this study and other reference strains are shown using a heat map with the results of their full genome similarity analysis displayed on the lower left (various identity values are expressed in gradient colors ranging from 88% to 100%). (b) in the evolutionary tree for the genome, the canine strains are marked with red pentagrams, feline strains with red squares, canine reference strains with blue pentagrams, and feline hosts with blue squares.

Based on the contig-assembled sequences, the whole genome of these GyVg1 strains was identified to have a full length of 2375 nucleotides (nt). The genome nt identity of the nine GyVg1 strains ranged from 96.17% (DOG01 and DOG06) to 99.2% (DOG03 and DOG06). Compared with the reference GyVg1 strains, DOG06 and RS/BR/2015 (accession NO.: KY039279, originated from chicken, Brazil, 2015) had the lowest similarity (90.53%), whereas DOG04, HE1511 (accession NO.: KX708514, originated from chicken, China, 2015), HLJ1603-1 (accession NO.: KX708520, originated from chicken, China, 2016), and HLJ1603-2 (accession NO.: KX708521, originated from chicken, China, 2016) had the highest similarity (99.2%). A similarity comparison of the obtained and reference strains is shown *via* a heatmap in Figure 1a.

**Figure 2.**
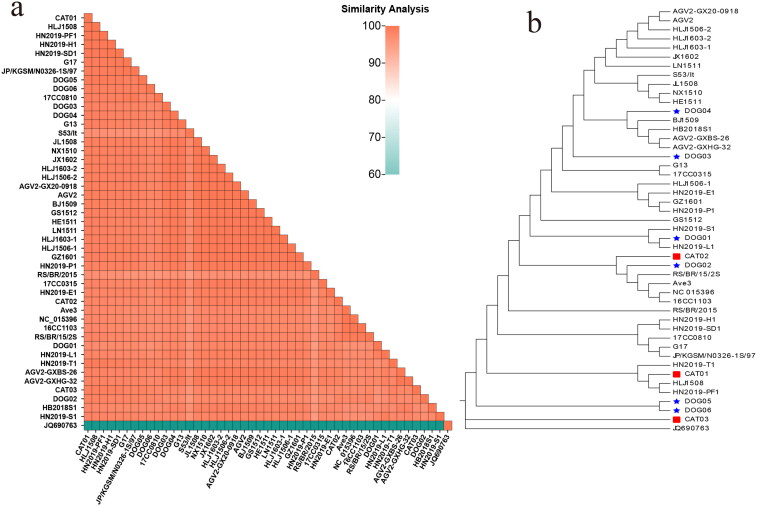
Analysis of the evolution and identity of the VP1 aa sequences. (a) Heat map showing the similarity analysis between the GyVg1 strains acquired in this study and other reference strains. The gradient colors on the lower left indicate different identity values ranging from 80% to 100%. (b) In the VP1 evolutionary tree, the canine strains are identified with red pentagrams, feline strains with red squares, canine hosts in the reference strains with blue pentagrams, and feline hosts with blue squares.

The VP1 aa sequences of the nine GyVg1 strains ranged from 96.09% (DOG01 and DOG02) to 99.35% (DOG03 and DOG 04). Compared with the reference strains, DOG01 and RS/BR/2015 (accession NO.: KY039279, originated from chicken, Brazil, 2015) had the lowest VP1 aa similarity of 95%, whereas CAT01, HLJ1508 (accession NO.: KX708511, originated from chicken, China, 2015), and HN2019-PF1 (accession NO.: OK540283, originated from peafowl, China, 2019) shared the highest similarity of 100. A similarity comparison of the VP1 aa sequence of the obtained and reference strains is shown in Figure 2a.

**Figure 3.**
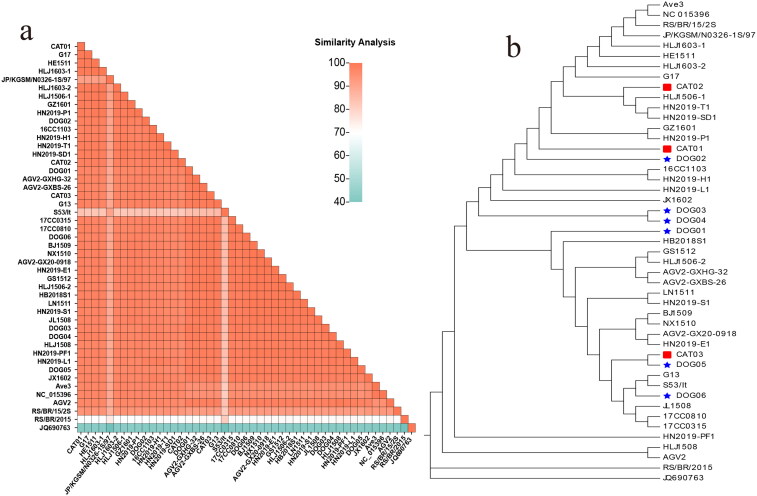
Analysis of the evolution and identity of the UTR nt sequences. (a) Heat map showing the comparison of similarity between the UTRs of the GyVg1 strains used in this study and other reference strains (bottom left: levels of identity are shown by gradient colors ranging from 40% to 100%).(b) in the evolutionary tree, the canine strains are marked with red pentagrams, feline strains with red squares, canine reference strains with blue pentagrams, and feline hosts with blue squares.

The nt similarity of the UTR between these nine strains ranged from 96.11% (DOG05 and CAT02) to 99.59% (DOG03 and DOG04). Compared with the reference strain, the nt similarity in the UTR ranged from 69.31% (CAT02 and BR/BR/2015) to 99.59% (DOG06 and the five reference strains consisting of G13 (accession NO.: KJ452214, originated from ferret, Hungary, 2011), NX1510 (accession NO.: KX708513, originated from chicken, China, 2015), BJ1509 (accession NO.: KX708512, originated from chicken, China, 2015), HN2019-E1 (accession NO.: OK540279, originated from egret, China, 2019), and AGV2-GX20-0918 (accession NO.: MW579760, originated from chicken, China, 2020). The nt similarity comparison of the UTR of all obtained and reference strains is shown in Figure 3a.

### Phylogenetic and recombination analyses

Phylogenetic analysis of the entire genome sequences revealed that the 9 obtained and the 38 reference GyVg1 strains were mainly clustered into three branches (Figure 1b). CAT03, DOG05, and DOG06 were clustered into a broad branch. DOG03, DOG04, and CAT02 were clustered in another branch. The remaining strains were dispersed on various subbranches of another branch. Similar to the phylogenetic tree of the entire genome, CAT03, DOG05, and DOG06 were distributed relatively close to the aa evolution tree of VP1. CAT02 and DOG02 were relatively scattered throughout the entire gene evolution tree but were distributed into one branch of the evolution tree based on the VP1 aa sequence (Figure 2b). In contrast, the obtained and reference strains were distributed into a wide branch on the UTR sequence evolution tree (Figure 3b).

**Figure 4.**
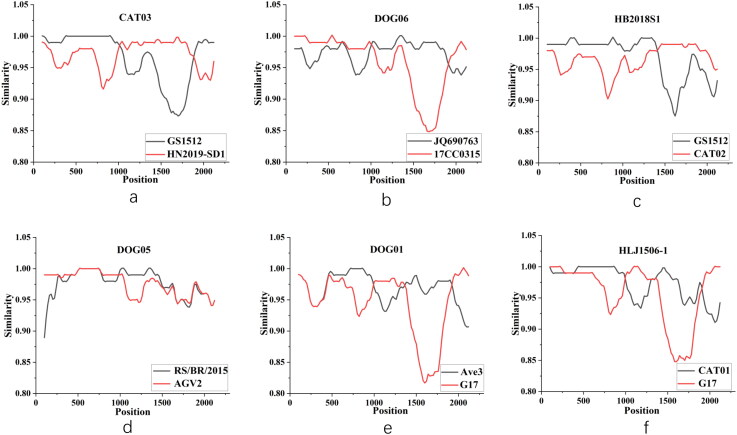
Recombination occurrence in CAT03, DOG06, CAT02, DOG05, DOG01, and CAT01 strains was predicted using simplot software. (a) Recombination event related to CAT03. (b) Recombination event related to DOG06. (c) Recombination event related to CAT02. (d) Recombination event related to DOG05. (e) Recombination event related to DOG06. (f) Recombination event related to CAT01.

The genome sequences of the obtained and reference strains were analyzed to determine the likelihood of genotype recombination. The results revealed six (CAT03, DOG06, CAT02, DOG05, DOG01, and CAT01) potential recombination events with high confidence values based on at least five independent algorithms (Table 2). The breakpoints predicted *via* the bootstrap approach and the parent strains for each recombinant strain are shown in Figure 4. Furthermore, DOG06 was a recombination from the major parent of JQ690763 (accession NO.: JQ690763, originated from human, China, 2012) and the minor parent of 17CC0315 (accession NO.: MK089244, originated from cat, China, 2017), whereas DOG05 was a recombination from the major parent AGV2 (accession NO.: MT671981, originated from chicken, 2018) and the minor parent RS/BR/2015 (accession NO.: KY039279, originated from chicken, 2015), both of which are from Brazil. DOG01 was a recombination from the major parent AVE3 (accession No.: HM590588, originated from chicken, 2006, Brazil) and the minor parent ferret G17 (accession No.: KY039279, originated from ferret, 2011, Hungary).

**Table 2.** Recombinant events predicted using different algorithms.

Event	RDP	GENECONV	BootScan	MaxChi	Chimaera	SiScan	3Seq
I	6.59E-08	2.37E-05	8.09E-06	7.20E-10	ns	1.05E-13	8.20E-24
II	2.34E-11	8.84E-10	3.99E-03	3.19E-08	1.73E-08	1.92E-10	6.99E-19
III	1.34E-04	2.57E-06	5.08E-06	5.66E-08	3.41E-06	1.67E-16	4.49E-15
IV	1.30E-05	2.93E-08	1.36E-07	1.15E-04	6.61E-05	2.85E-05	6.88E-07
V	7.28E-05	1.71E-03	ns	1.14E-03	0.025924	2.91E-02	ns

### Mutation analysis

For GyVg1, ORF3 encodes VP1, located at 953–2335 in the genome, and has 460 aa residues. Compared with the reference strains from different hosts, the nine obtained strains contained 30 substituted amino acids in VP1 (displayed in Supplementary Table S2), comprising nine unique amino acid mutations at sites 12 R/H, 114S/N, 123I/M, 167 L/P, 231 P/S, 237 P/L, 243 R/W, 335 T/A, and 444S/N, whereas 53K/R and 105 G/V were only consistent with JQ690763, which originated from humans. ORF1 encoded 231 aa sequences of VP2 for GyVg1. There were 18 altered amino acid sites harbored in the obtained strains (displayed in Supplementary Table S3), while 81 A/P, 103 R/H, 223 R/G, and 228 A/T were the only mutations compared with other reference strains of different hosts. Additionally, 14 N/T, 156 G/R, 157K/R, 158 R/G, and 180 A/V were similar to AGV2-GXBS-26, which originated from the dog. VP3, a nonstructural protein with 124 aa residues, was encoded by ORF2. Compared with the reference strains, the obtained strains had specific aa substitutions at sites 38 M/I, 61 A/T, and 65 V/A. 71E/G in DOG05 was compared with JQ690763. The following were the other notable aa mutation sites: 9 R/Q, 14Q/R, 79 V/P/A, 99 A/S, 103K/R, 104Q/R, and 115 N/E compared with AGV2-GXBS-26 (displayed in Supplementary Table S4).

## Discussion

As previously mentioned, GyVg1 DNA has been detected in various organisms; however, for collecting sufficient evidence regarding its infection and transmission, successful isolation and artificial infection experiments need to be performed. In this study, GyVg1 DNA was detected in the sera of cats and dogs. However, GyVg1 has not been isolated from common cells originating from chickens, cats, or dogs, which is similar to the findings of other studies (Niu et al. 2019). This could be because of the specific viral tropism, and the used cell line was not suitable for GyVg1 infection or replication. Therefore, serological detection assay for GyVg1 should be developed and sero-epidemiological investigation may provide insight in seroprevalence and occurrence of the virus in domestic animals. According to the evolutionary tree for the entire genome, GyVg1 strains identified in dogs and cats in this study were dispersed from other reference stains from different regions or hosts. Although the branches of evolutionary trees for VP1 and UTR differed, they were still not determined by host and region. Combining the similarity and differential distribution of strains in these evolutionary trees indicated that the evolution complexity may be caused by the wide host spectrum of GyVg1, suggesting the recombination occurrence of GyVg1.

Recombination is considered an important trend for gyrovirus evolution, which has been predicted in most of the obtained GyVg1 strains (Tan et al. 2020; Ji et al. 2022). CAT03 was recombined from the major parent of HN2019-SD1 (identified in sika deer, China, 2019) and the minor parent of GS1512 (identified in chicken, China, 2015). DOG05 was recombined from the major parent of AGV2 (identified in chicken, Brazil, 2018) and the minor parent of RS/BR/2015 (identified in chicken, Brazil, 2015). DOG06 was recombined from the human-originated JQ690763 strain and dog-originated AGV2-GXHG-32 strain. DOG01 was recombined from the major parent of Ave3 (identified in chicken, Brazil, 2006) and the minor parent of G17 (identified in ferret, Hungary, 2011). In summary, the predicted recombination event was related to strains detected in chickens, dogs, and cats as well as those in humans and ferrets, suggesting a more complex horizontal transmission of GyVg1.

As the capsid of GyVg1, VP1 may play a key role in receptor binding and virus–host interaction. Based on a previous report, GyVg1-VP1 contained a hypervariable region with residues varying from 288 to 314 aa. (Yao et al. 2016). Most of the obtained strains had mutations in the hypervariable region, but the mutation sites were not the same. Further studies are necessary to determine whether this mutant region affects host adaption of GyVg1. The VP2 protein of GyVCA is reportedly a dual-specific protein phosphatase (Peters et al. 2002). Furthermore, the ‘WX7HX3CXCX5H’ sequence of the phosphatase motif is highly conserved in GyVCA, Torque Teno virus (TTV), and TTV-like mini virus (Takahashi et al. 2000). Similar to GyVCA, ‘WLRQCARSHDEICTCGRWRSH’ (95–115 residues) is a comparable motif that performs VP2 site-directed mutagenesis and is related to impairing viral particle replication, which has also been found in GyVg1 VP2 sequences (Peters et al. 2002). Although GyVCA-VP2 is a nonstructural protein, it has been shown to serve a scaffold function in the assembly of GyVCA particles by interacting with the VP1 and VP2 proteins (Noteborn et al. 1998). The VP2 protein of the obtained strains had 18 representative aa mutations, of which 10 were unique to these strains. Further research is necessary to determine whether such mutations impact the scaffold function of the VP2 protein and whether they alter the VP2 function associated with virus propagation.

Similar to GyVCA, the VP3 protein of GyVg1 can particularly induce apoptosis in tumor cells (Bullenkamp et al. 2012). The GyVg1 strains in this study also contained multiple mutations in the VP3 protein loci 28S/C, 38 M/I, 54S/Y, 61 A/T, 65 V/A, 69 A/D, and 81S/L. More experimental evidence is needed to determine whether these mutations are responsible for the differences in the molecular function of GyVg1 VP3.

In this study, nine GyVg1 strains were identified in the sera of companion animals. Phylogenetic, recombination, and mutation analyses provided more information for a better understanding of the transmission manner and host variety of GyVg1.

## Supplementary Material

Supplemental Material

## Data Availability

All data generated or analyzed during this study are included in this article.
