# Supplementary material for: Molecular analysis of Gyrovirus galga1 variants identified from the sera of dogs and cats in China
Source: Vet Q. 2024 Apr 10;44(1):1–8. doi: 10.1080/01652176.2024.2338381 (PMC11008310; doi:10.1080/01652176.2024.2338381)
Supplement: Supplemental Material [file TVEQ_A_2338381_SM0854.docx]

Supplementary Table 1**.** Detailed information the reference stains used in this study.

| **Strain name** | **Virus species** | **Accession No.** | | **Year** | **Country** | **Host** |
| --- | --- | --- | --- | --- | --- | --- |
| Ave 3 | GyVg1 | HM590588 | 2006 | | Brazil | Chicken |
| JQ690763 | GyVg1 | JQ690763 | 2012 | | China | human |
| G17 | GyVg1 | KJ452213 | 2011 | | Hungary | ferret |
| G13 | GyVg1 | KJ452214 | 2011 | | Hungary | ferret |
| S53/It | GyVg1 | KU168250 | 2014 | | China | Chicken |
| HLJ1506–1 | GyVg1 | KX708506 | 2015 | | China | Chicken |
| HLJ1508 | GyVg1 | KX708510 | 2015 | | China | Chicken |
| JL1508 | GyVg1 | KX708511 | 2015 | | China | Chicken |
| NX1510 | GyVg1 | KX708513 | 2015 | | China | Chicken |
| HE1511 | GyVg1 | KX708514 | 2015 | | China | Chicken |
| LN1511 | GyVg1 | KX708515 | 2015 | | China | Chicken |
| GS1512 | GyVg1 | KX708517 | 2015 | | China | Chicken |
| JX1602 | GyVg1 | KX708519 | 2016 | | China | Chicken |
| HLJ1603-1 | GyVg1 | KX708520 | 2016 | | China | Chicken |
| HLJ1603-2 | GyVg1 | KX708521 | 2016 | | China | Chicken |
| HLJ1506-2 | GyVg1 | KX708522 | 2015 | | China | Chicken |
| GZ1601 | GyVg1 | KX708518 | 2016 | | China | Chicken |
| JP/KGSM/N0326-1S/97 | GyVg1 | LC716408 | 1997 | | Japan | Chicken |
| AGV2-GX20-0918 | GyVg1 | MW579760 | 2020 | | China | Chicken |
| HB2018S1 | GyVg1 | MK840982 | 2018 | | China | Snake |
| 17CC0810 | GyVg1 | MK089246 | 2017 | | China | Cat |
| NC_015396 | GyVg1 | NC_015396 | 2006 | | Brazil | Chicken |
| RS/BR/15/2S | GyVg1 | MG846492 | 2018 | | Brazil | Chicken |
| 17CC0315 | GyVg1 | MK089244 | 2017 | | China | Cat |
| 16CC1103 | GyVg1 | MK089245 | 2016 | | China | Cat |
| AGV2-GXBS-26 | GyVg1 | OK245348 | 2019 | | China | Dog |
| AGV2-GXHG-32 | GyVg1 | OK245349 | 2019 | | China | Dog |
| HN2019-E1 | GyVg1 | OK540279 | 2019 | | China | Egret |
| HN2019-S1 | GyVg1 | OK540280 | 2019 | | China | Silver Pheasant |
| HN2019-H1 | GyVg1 | OK540281 | 2019 | | China | Hippopotamus |
| HN2019-T1 | GyVg1 | OK540282 | 2019 | | China | Tiger |
| HN2019-PF1 | GyVg1 | OK540283 | 2019 | | China | Peafowl |
| HN2019-SD1 | GyVg1 | OK540284 | 2019 | | China | Sika deer |
| HN2019-L1 | GyVg1 | OK540285 | 2019 | | China | Lion |
| HN2019-P1 | GyVg1 | OK540286 | 2019 | | China | Pheasants |
| AGV2 | GyVg1 | MT671981 | 2018 | | Brazil | Chicken |
| BJ1509 | GyVg1 | KX708512 | 2015 | | China | Chicken |
| RS/BR/2015 | GyVg1 | KY039279 | 2015 | | Brazil | Chicken |

Supplementary Table 2**.** Mutations at different amino acid sites in GyVg1 VP1.

| **Strain** | **Host** | **Substitution of the Amino Acid Residues in VP2** | | | | | | | | | | | | | | | | | | | | | | | | | | | | | |
| --- | --- | --- | --- | --- | --- | --- | --- | --- | --- | --- | --- | --- | --- | --- | --- | --- | --- | --- | --- | --- | --- | --- | --- | --- | --- | --- | --- | --- | --- | --- | --- |
|  |  | 12 | 36 | 44 | 53 | 59 | 96 | 105 | 114 | 123 | 158 | 161 | 167 | 231 | 237 | 243 | 246 | 256 | 257 | 272 | 273 | 293 | 298 | 315 | 319 | 326 | 335 | 388 | 408 | 423 | 444 |
| Consensus site |  | R | G | R | R | H | K | V | N | M | S | M | P | S | L | W | R | A | P | N | V | Q | Q | E | R | C | A | P | V | L | N |
| CAT01 | cat | - | - | - | - | - | T | - | - | - | - | - | - | - | - | - | - | - | - | - | - | - | - | - | - | - | - | - | - | - | - |
| DOG01 | dog | - | S | - | - | R | - | - | - | - | A | - | L | P | - | - | K | V | - | D | M | V | G | - | - | - | - | - | - | - | - |
| CAT02 | cat | - | - | - | - | - | - | - | - | - | A | - | - | - | - | - | - | - | - | - | - | V | G | - | - | S | - | Q | M | I | - |
| DOG02 | dog | - | - | - | - | - | T | - | - | - | - | - | - | - | - | - | - | - | S | - | - | - | - | - | K | - | T | Q | M | I | - |
| DOG03 | dog | - | - | - | - | - | - | - | - | I | A | - | - | - | - | R | - | - | - | - | - | V | G | - | - | - | - | - | - | - | - |
| DOG04 | dog | H | - | - | - | - | - | - | - | - | A | - | - | - | - | - | - | - | - | - | - | V | G | - | - | - | - | - | - | - | - |
| CAT03 | cat | - | - | - | K | - | T | G | - | - | - | K | - | - | - | - | - | - | - | - | - | - | - | G | - | - | - | - | - | - | - |
| DOG05 | dog | - | - | - | - | - | T | - | - | - | - | - | - | - | P | - | - | - | - | - | - | - | - | - | - | - | - | - | - | - | S |
| DOG06 | dog | - | - | K | - | - | T | - | S | - | - | - | - | - | - | - | - | - | - | - | - | - | - | - | K | - | - | - | - | - | S |
| Ave3 | chicken | - | - | - | - | - | T | - | - | - | - | - | - | - | - | - | G | - | - | - | - | V | G | Q | - | - | - | Q | M | I | - |
| JQ690763 | human | - | Y | Q | K | R | A | G | - | - | C | P | - | - | - | - | - | Q | N | Y | A | I | T | M | Q | T | - | A | K | E | - |
| G17 | ferret | - | S | - | - | - | - | - | - | - | - | - | - | - | - | - | - | - | - | - | - | - | - | - | - | - | - | - | - | - | - |
| 17CC0810 | cat | - | S | - | - | - | - | - | - | - | - | - | - | - | - | - | G | - | - | - | - | - | - | - | - | - | - | - | - | - | - |
| AGV2-GXBS-26 | dog | - | G | X | - | - | - | - | - | - | A | - | - | - | - | - | - | - | - | - | - | V | G | - | - | - | - | - | - | - | - |

“-” site of obtained strain same as the Consensus site.

Supplementary Table 3**.** Mutations at different amino acid sites in GyVg1 VP2.

| **Strain** | **Host** | | **Substitution of the Amino Acid Residues in VP2** | | | | | | | | | | | | | | | | | |
| --- | --- | --- | --- | --- | --- | --- | --- | --- | --- | --- | --- | --- | --- | --- | --- | --- | --- | --- | --- | --- |
|  |  |  | 14 | 74 | 81 | 103 | 124 | 141 | 156 | 157 | 158 | 161 | 165 | 175 | 176 | 180 | 182 | 214 | 223 | 228 |
| Consensus site | |  | T | R | P | H | V | Q | R | R | G | H | T | D | D | V | A | D | G | T |
| CAT01 | | cat | - | - | - | - | - | - | - | - | - | - | - | - | - | - | - | - | - | - |
| DOG01 | | dog | - | G | - | - | - | - | - | - | - | - | - | - | - | - | - | - | - | A |
| CAT02 | | cat | - | - | A | - | - | - | - | - | - | - | - | - | - | - | - | - | - | - |
| DOG02 | | dog | - | - | - | - | - | - | - | - | - | - | - | - | - | - | - | - | - | - |
| DOG03 | | dog | - | - | - | - | - | - | - | - | - | - | - | - | - | - | - | - | - | - |
| DOG04 | | dog | - | - | - | - | I | - | - | - | - | - | - | - | - | - | T | - | - | - |
| CAT03 | | cat | N | - | - | - | - | R | G | K | R | Y | A | E | E | A | - | - | R | - |
| DOG05 | | dog | N | - | - | - | - | - | - | - | - | - | A | - | - | - | - | - | - | - |
| DOG06 | | dog | N | - | - | R | - | R | G | K | R | Y | A | E | E | A | - | N | - | - |
| Ave3 | | chicken | - | - | - | - | - | - | - | - | - | - | - | - | - | - | - | - | - | - |
| JQ690763 | | human | Q | - | - | - | E | R | S | Q | P | N | V | L | A | A | F | S | - | - |
| G17 | | ferret | - | N | - | - | - | R | - | - | - | - | - | - | - | A | - | - | - | - |
| 17CC0810 | | cat | - | - | - | - | - | R | G | K | - | - | - | - | - | A | - | - | - | - |
| AGV2-GXBS-26 | | dog | N | - | - | - | - | R | G | K | R | - | - | - | - | A | - | X | - | - |

“-” site of obtained strain same as the Consensus site.

Supplementary Table 4**.** Mutations at different amino acid sites in GyVg1 VP3.

| **Strain** | **Host** | **Substitution of the Amino Acid Residues in VP3** | | | | | | | | | | | | | | | |
| --- | --- | --- | --- | --- | --- | --- | --- | --- | --- | --- | --- | --- | --- | --- | --- | --- | --- |
|  |  | 9 | 14 | 28 | 38 | 54 | 61 | 65 | 69 | 71 | 79 | 81 | 99 | 101 | 103 | 104 | 115 |
| Consensus site |  | Q | R | C | I | Y | T | A | D | G | A | L | S | K | R | R | E |
| CAT01 | cat | - | - | - | - | - | - | - | - | - | - | S | - | - | - | - | - |
| DOG01 | dog | - | - | - | - | - | - | - | - | - | - | S | - | - | - | - | - |
| CAT02 | cat | - | - | - | M | - | - | - | - | - | - | S | - | - | - | - | - |
| DOG02 | dog | - | - | - | - | - | - | - | - | - | - | S | - | - | - | - | - |
| DOG03 | dog | - | - | - | - | - | - | - | - | - | - | S | - | - | - | - | - |
| DOG04 | dog | - | - | - | - | - | - | - | - | - | - | S | - | - | - | - | - |
| CAT03 | cat | R | Q | S | - | - | - | - | - | - | V | - | A | - | K | Q | N |
| DOG05 | dog | R | Q | - | - | S | - | V | A | E | V | - | - | R | K | - | - |
| DOG06 | dog | R | Q | S | - | - | A | - | - | - | V | - | A | - | K | Q | N |
| Ave3 | chicken | - | - | - | - | - | - | - | - | - |  | S | - | - | - | - | - |
| JQ690763 | human | L | P | R | - | C | - | - | N | E | P | - | E | R | S | E | S |
| G17 | ferret | - | - | - | - | - | - | - | - | - | V | - | A | - | K | - | - |
| 17CC0810 | cat | - | Q | - | - | - | - | - | - | - | V | - | A | - | - | - | K |
| AGV2-GXBS-26 | dog | R | Q | - | - | - | - | - | - | - | V | - | A | - | K | Q | N |

“-” site of obtained strain same as the Consensus site.
